# Prognostic values of signal transducers activators of transcription in gastric cancer

**DOI:** 10.1042/BSR20181695

**Published:** 2019-04-30

**Authors:** Yujie Zhang, Chaoran Yu

**Affiliations:** 1 Department of Gastrointestinal Surgery, Tongji Hospital, Tongji Medical College, Huazhong University of Science and Technology, Wuhan, 430030, China; 2 Fudan University Shanghai Cancer Center, Fudan University, Shanghai 200025, P.R. China; 3 Department of General Surgery, Ruijin Hospital, Shanghai Jiao Tong University School of Medicine, Shanghai 200025, P.R. China; 4 Shanghai Minimally Invasive Surgery Center, Ruijin Hospital, Shanghai Jiao Tong University School of Medicine, Shanghai, 200025, P.R. China

**Keywords:** Gastric cancer, KM plotter, Prognosis, STATs

## Abstract

The signal transducers and activators of transcription genes family (STATs) have been well studied as prognostic predictors for various solid tumors, but their prognostic values in gastric cancer (GC) patients have not been fully elucidated. The ‘Kaplan–Meier plotter’ and multiple public available databases were used for the characterization of the prognostic roles of STATs family in GC. The results indicated that high mRNA expression of all individual STATs, except STAT3 and STAT6, were significantly associated with favorable overall survival (OS) in GC. Moreover, the prognostic values of STATs were further characterized in subtypes, including HER2 status, Lauren’s classification, differentiation, and clinical stages. Moreover, the prognostic value of STATs signature was also characterized. Low risk group displayed a significantly favorable OS than high risk (HR: 1.71; 95% CI: 1.09–2.66, *P*=0.0184). In addition, STATs showed distinct expression between GC and normal groups. Meanwhile, comparable high correlation between STATs and tumor immune infiltrating cells (TIICs) was also observed. STAT4 displayed highest correlation with dendritic cells (correlation = 0.716, *P*=1.63e-59) and CD8^+^ T cells (correlation = 0.697, *P*=5.02e-55). In conclusion, our results suggest that all individual STATs, except STAT3 and STAT6, may act as prognostic markers in GC.

## Introduction

Gastric cancer (GC) is the fourth most common cancer and second leading cause of cancer-related deaths in the world, accounting for approximate 9% of total cancer deaths [1,2]. Although the 5-year overall survival (OS) can reach more than 90% in early GC by early diagnosis and multi-disciplinary therapeutic strategies, the prognosis of advanced or metastatic GC patients remain largely unsatisfactory, with median survival period being around 1 year [3,4]. Therefore, novel prognostic biomarkers for GC could contribute to the identification of those risky cases and maximize the OS benefits.

Signal transducers and activators of transcription (STATs) are a gene family of cytoplasmic transcription factors, consisting of seven members, STAT1–STAT4, STAT5a, STAT5b, and STAT6 [5,6]. STATs play important roles in numerous biological processes, including cell proliferation, differentiation, apoptosis, and survival [7]. STATs are activated via tyrosine phosphorylation, a process which occurs either through KIT-based interaction or cytokine-induced JAK pathway [8]. STATs can also be activated by constitutively activated non-receptor protein tyrosine kinases (PTKs), including c-Src and Bcr-Abl. Activated STATs rapidly translocate into nucleus, and bind to the promoter region of target genes, serving as transcription regulators. Increasing evidence have indicated that the STATs, particularly STAT1, STAT3, and STAT5 play critical roles in various cancer progressions and have been identified as potential therapeutic targets [9–13]. Nevertheless, reports focusing on the relationship between GC and STATs remain limited [14–16]. In fact, the prognostic values of STATs family in GC patients are yet to be fully characterized.

In the present study, we comprehensively explored the prognostic values of seven STATs genes using the Kaplan–Meier (KM) plotter online database and multiple public available databases.

## Materials and methods

### KM plotter prognostic analysis

The prognostic values of STATs family (STAT1, STAT2, STAT3, STAT4, STAT5a, STAT5b, and STAT6) in GC were investigated in KM Plotter, an online database (http://kmplot.com/analysis/) providing prognostic evaluation of selected genes on breast, ovarian, lung, and GC patients [17]. Corresponding GC datasets used in KM plotter were retrieved from Gene Expression Omnibus (GEO), including GSE14210, GSE15459, GSE22377, GSE29272, GSE51105 and GSE62254. In addition, differentiation, human epidermal growth factor receptor 2 (HER2) status, Lauren classification, and TNM stage data were also obtained for subtype analysis. Briefly, the cutoff values for individual STAT genes expression between ‘low’ and ‘high’ were determined by optimal cutoff algorithm. The prognostic values between high and low expression groups were evaluated by a KM curve with hazard ratio (HR), 95% confidence intervals (CI), and log-rank *P*-value. *P*-value <0.05 was considered to be statistically significant.

### Prognostic values of STATs signature via the SurvExpress platform

The prognostic values of STATs signature were explored via the SurvExpress platform (http://bioinformatica.mty.itesm.mx:8080/Biomatec/SurvivaX.jsp) with the stomach adenocarcinoma (STAD) of the cancer genome atlas (TCGA) selected as input dataset (*n*=352) [18]. High/low risk groups were categorized based on the default prognostic risk algorithm [18].

### Oncomine database analysis

The mRNA expression of STATs members was investigated via the Oncomine database, which was a comprehensive cancer genomic platform [19]. Briefly, the mRNA expression of each STAT gene was compared between cancer and normal groups with *P-*value <0.05 set as statistically significant. Only significant results were displayed.

### Tumor immune infiltrating cells (TIICs) correlation analysis

The correlations between each STAT and tumor immune infiltrating cells (TIICs) (B cells, CD4^+^ T cells, CD8^+^ T cells, dendritic cells, macrophages, and neutrophils) were analyzed via the Tumor IMmune Estimation Resource (TIMER) platform (https://cistrome.shinyapps.io/timer/) [20]. The correlation was further corrected by the tumor purity-based Spearman’s method [20].

## Results

### Prognostic values of STATs in all GC patients

**Figure 1:**
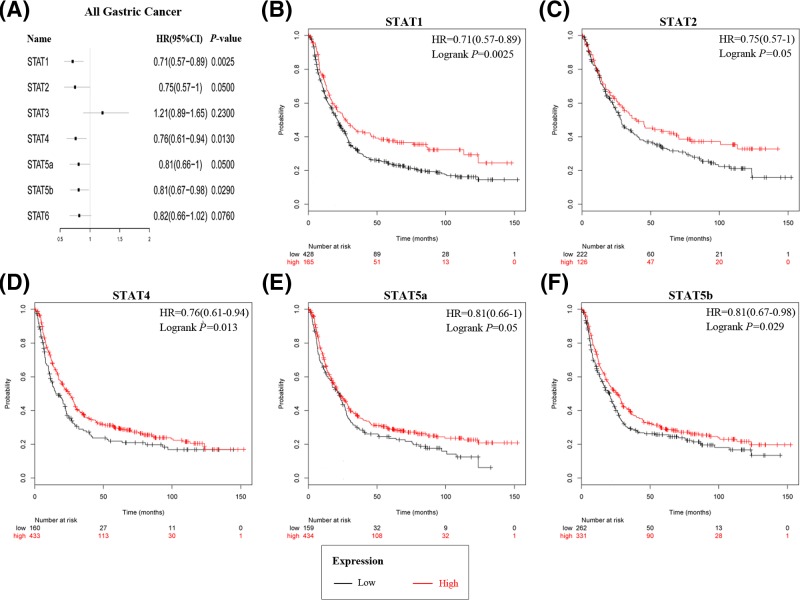
The prognostic values of individual STATs mRNA level in all GC patients (**A**) Forest plot for the relationship between individual STATs mRNA expression and prognostic OS in all GC patients. OS curves of (**B**) STAT1 (Affymetrix ID: 200887_s_at), (**C**) STAT2 (Affymetrix ID: 225636 _at), (**D**) STAT4 (Affymetrix ID: 206118 _at), (**E**) STAT5a (Affymetrix ID: 203010 _at), and (**F**) STAT5b (Affymetrix ID: 212549 _at) are plotted for all GC patients.

The prognostic values of STATs in all GC were investigated via the KM plotter. Among seven STATs members, five were significantly associated with prognosis for all GC patients (Figure 1A). High mRNA expression of STAT1 (HR: 0.71; 95% CI: 0.57–0.89; *P*=0.0025), STAT2 (HR: 0.75; 95% CI: 0.57–1; *P*=0.05), STAT4 (HR: 0.76; 95% CI: 0.61–0.94; *P*=0.013), STAT5a (HR: 0.81; 95% CI: 0.66–1; *P*=0.05), and STAT5b (HR: 0.81; 95% CI: 0.67–0.98; *P*=0.029) were associated with better OS (Figure 1B–F). STAT6 (HR: 0.82; 95% CI: 0.66–1.02; =0.076) and STAT3 (HR: 1.21; 95% CI: 0.89–1.65; *P*=0.23) did not show significant prognostic values (Figure 1A).

### Prognostic values of STATs in HER2 subtypes

**Figure 2:**
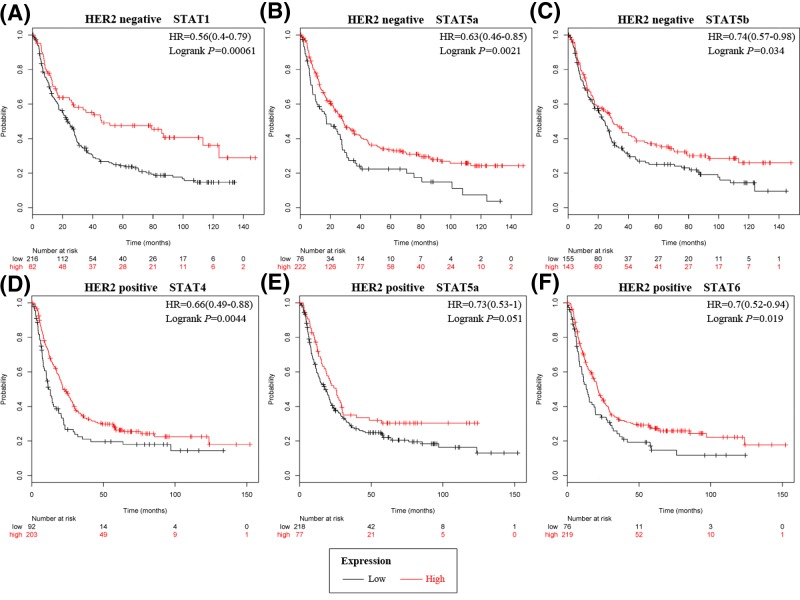
The prognostic values of individual STATs mRNA level with HER2 gene OS curves of (**A**) STAT1, (**B**) STAT5a, and (**C**) STAT5b are plotted in GC patients with HER2-negative gene. OS curves of (**D**) STAT4, (**E**) STAT5a, and (**F**) STAT6 (Affymetrix ID: 201331_s_at) are plotted in GC patients with HER2-positive gene.

Next, the prognostic values of STATs associated with the HER2 subtypes in GC patients were explored (Figure 2). High mRNA expression of STAT1, STAT5a, and STAT5b were associated with better OS in HER2-negative GC patients (Figure 2). Meanwhile, high expression of STAT4 and STAT6 were associated with better OS in HER2-positive GC patients. STAT5a was modestly associated with favorable OS in HER2-positive GC patients (Figure 2). The rest STAT members were not significantly correlated with prognosis.

### Prognostic values of STATs in GC patients with Lauren’s classification

**Figure 3:**
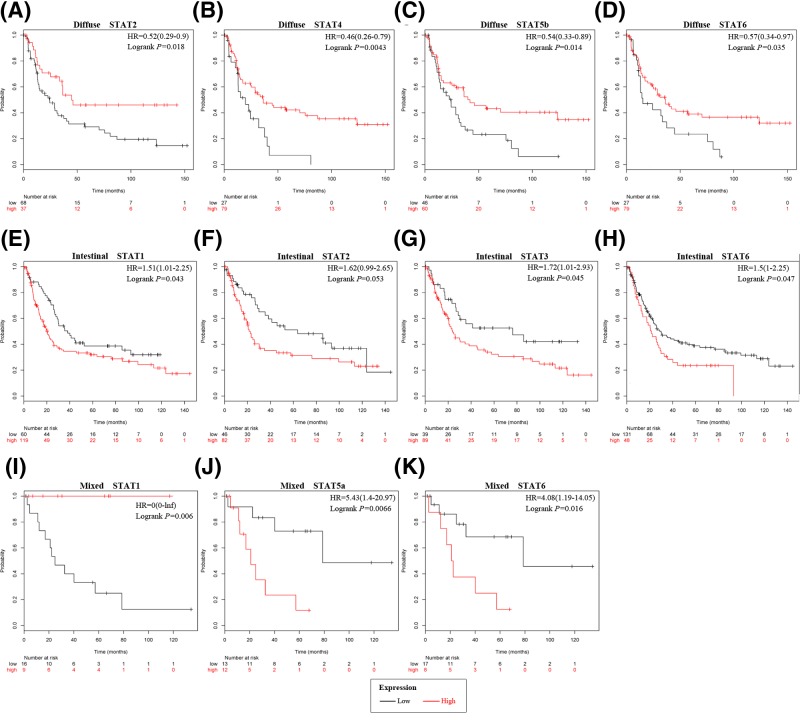
The prognostic values of individual STATs mRNA level in GC with histological subtypes according to Lauren’s classification OS curves of (**A**) STAT2, (**B**) STAT4, (**C**) STAT5b, and (**D**) STAT6 are plotted for diffuse type GC patients. OS curves of (**E**) STAT1, (**F**) STAT2, (**G**) STAT3 (Affymetrix ID: 225289 _at), and (**H**) STAT6 are plotted for intestinal type GC patients. OS curves of (**I**) STAT1, (**J**) STAT5a, and (**K**) STAT6 are plotted for mixed-type GC patients.

High mRNA expression of STAT2, STAT4, STAT5b, and STAT6 were associated with favorable OS in the diffuse type of GC patients (Figure 3A–D). High expression of STAT1, STAT3, and STAT6 mRNA expression in intestinal type GC patients were significantly associated with unfavorable OS, while STAT2 was modestly associated with unfavorable OS (Figure 3E–H). High expression of STAT1 mRNA was associated with favorable OS in mixed type GC patient whereas STAT5a and STAT6 were associated with unfavorable OS (Figure 3I–K).

### Prognostic values of STATs in GC patients with differentiation subtypes

**Figure 4:**
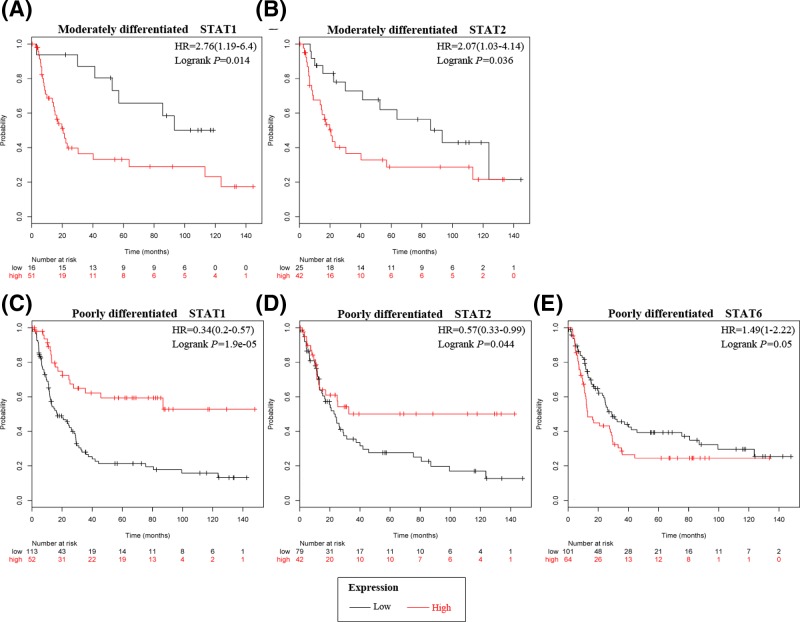
The prognostic values of individual STATs mRNA level in GC patients with various differentiation OS curves of (**A**) STAT1 and (**B**) STAT2 are plotted for moderately differentiated type GC patients. OS curves of (**C**) STAT1, (**D**) STAT2, and (**E**) STAT6 are plotted for poorly differentiated type GC patients.

High mRNA expression of STAT1 and STAT2 were associated with unfavorable prognosis in moderately differentiated type GC, but with favorable prognosis in poorly differentiated type GC (Figure 4A–D). High expression of STAT6 was associated with unfavorable OS in poorly differentiated type GC (Figure 4E).

### Prognostic values of STATs in different GC clinical stages

Only high expression of STAT4 was associated with worse prognosis in stage I in GC patients (Table 1). In stage III, high expression of STAT1, STAT2, and STAT4 were associated with favorable OS, while STAT5b was associated with poor OS (Table 1). None of the STAT members were found to be correlated with OS in stages II and IV. In addition, high expression of STAT6 was associated with worse prognosis in lymph node-negative GC. For lymph node-positive GC, STAT1 and STAT3 were associated with worse survival, whereas STAT4 was associated with better OS (Table 1). High level of STAT1 mRNA expression was associated with worse prognosis both in M0 and M1 stage GC patients whereas STAT3 was associated with poor OS in M0 stage (Table 1).

**Table 1:** Prognostic correlation of STATs mRNA expression in GC patients with different clinical stage

Clinical stage	STATs	Cases	HR (95% CI)	*P*-value
Stage I				
	STAT1	39	0.63 (0.21–1.88)	0.4
	STAT2	34	0.4 (0.08–1.95)	0.24
	STAT3	34	0.34 (0.07–1.61)	0.15
	STAT4	39	4.32 (1.41–13.21)	0.0055*
	STAT5a	39	0.37 (0.12–1.17)	0.079
	STAT5b	39	0.41 (0.12–1.35)	0.13
	STAT6	39	2.24 (0.74–6.8)	0.14
Stage II				
	STAT1	49	2.45 (0.72–8.36)	0.14
	STAT2	44	0.48 (0.14–1.67)	0.24
	STAT3	44	2.17 (0.49–9.55)	0.3
	STAT4	49	0.5 (0.21–1.17)	0.1
	STAT5a	49	1.58 (0.67–3.71)	0.29
	STAT5b	49	1.69 (0.57–4.99)	0.34
	STAT6	49	2.53 (0.74–8.69)	0.13
Stage III				
	STAT1	217	0.61 (0.41–0.9)	0.011*
	STAT2	109	0.62 (0.39–1)	0.05*
	STAT3	109	0.82 (0.52–1.3)	0.4
	STAT4	217	0.64 (0.43–0.94)	0.021*
	STAT5a	217	0.87 (0.6–1.24)	0.43
	STAT5b	217	1.41 (1.02–1.94)	0.035*
	STAT6	217	1.13 (0.82–1.56)	0.45
Stage IV				
	STAT1	74	1.38 (0.8–2.41)	0.25
	STAT2	66	0.64 (0.32–1.27)	0.2
	STAT3	66	1.65 (0.87–3.13)	0.12
	STAT4	74	0.63 (0.33–1.19)	0.15
	STAT5a	74	1.29 (0.75–2.23)	0.36
	STAT5b	74	0.79 (0.46–1.37)	0.4
	STAT6	74	0.74 (0.42–1.32)	0.31
LN (–)				
	STAT1	38	3.34 (0.75–14.78)	0.092
	STAT2	38	2.15 (0.78–5.94)	0.13
	STAT3	38	0.57 (0.21–1.56)	0.27
	STAT4	38	0.53 (0.19–1.46)	0.21
	STAT5a	38	0.42 (0.12–1.5)	0.17
	STAT5b	38	1.72 (0.64–4.6)	0.28
	STAT6	38	2.73 (0.99–7.57)	0.045*
LN (+)				
	STAT1	175	1.78 (1.15–2.75)	0.0083*
	STAT2	175	0.68 (0.44–1.05)	0.082
	STAT3	175	1.72 (1.14–2.6)	0.0095*
	STAT4	175	0.64 (0.44–0.94)	0.023*
	STAT5a	175	0.77 (0.52–1.14)	0.19
	STAT5b	175	0.78 (0.53–1.15)	0.22
	STAT6	175	1.42 (0.92–2.17)	0.11
M0				
	STAT1	186	1.74 (1.11–2.73)	0.015*
	STAT2	186	0.72 (0.45–1.15)	0.16
	STAT3	186	1.62 (1.05–2.5)	0.027*
	STAT4	186	0.67 (0.44–1)	0.051
	STAT5a	186	0.7 (0.47–1.03)	0.068
	STAT5b	186	0.69 (0.46–1.03)	0.066
	STAT6	186	1.3 (0.88–1.92)	0.19
M1				
	STAT1	31	3.86 (1.38–10.84)	0.0064*
	STAT2	31	2.37 (0.87–6.44)	0.082
	STAT3	31	1.85 (0.76–4.48)	0.17
	STAT4	31	2.8 (0.94–8.4)	0.056
	STAT5a	31	0.54 (0.23–1.31)	0.17
	STAT5b	31	1.42 (0.59–3.44)	0.43
	STAT6	31	2.33 (0.92–5.89)	0.066

* *P*<0.05.

### The prognostic value of STATs signature

**Figure 5:**
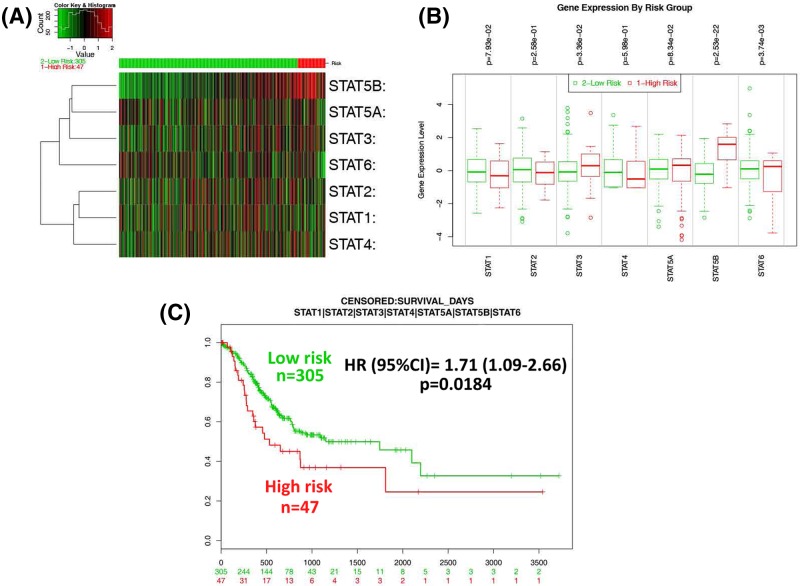
The prognostic values of STATs signature (**A**) Expression heat map between low (green, *n*=305) and high (red, *n*=47) risk groups; (**B**) expression comparison between low and high risk groups; (**C**) the survival analysis between low and high risk groups.

Intriguingly, low risk group displayed a significantly favorable OS than high risk (HR: 1.71; 95% CI: 1.09–2.66, *P*=0.0184) (Figure 5C), indicating potential prognostic role of STATs signature in GC. Of note, STAT5B showed the most up-regulated expression in high risk compared with low risk (Figure 5A, B).

### The mRNA expression analysis of STATs in GC

The expression levels of STATs have been investigated via the Oncomine database. Intriguingly, compared with normal group, STATs showed distinct expression not only in general GC, but also in Laurens subtypes (intestinal, mixed, and diffuse types) and pathological types (papillary adenocarcinoma and tubular adenocarcinoma) (Table 2).

**Table 2:** Comparison of transcription expression of STATs family members between different subtypes of GC and normal tissues via Oncomine

STAT members	Types of GC vs normal (N)	*P*-value	*T*-test	Fold change	Resources
STAT1	Intestinal type vs N	6.96E-15	9.751	2.703	[21]
	Mixed type vs N	1.34E-04	5.291	2.449	[21]
	GC vs N	5.48E-05	3.971	1.348	[22]
	Intestinal type vs N	0.002	3.081	1.881	[23]
	Diffuse type vs N	0.005	2.711	1.656	[23]
	Diffuse type vs N	0.006	3.282	1.854	[24]
	Intestinal type vs N	2.45E-06	5.146	1.996	[24]
	Mixed type vs N	0.006	4.459	5.034	[24]
	Intestinal type vs N	0.026	2.006	1.026	[19]
	Gastric adenocarcinoma vs N	2.84E-04	3.627	1.035	[25]
	Diffuse type vs N	0.008	2.501	1.016	[25]
	GC vs N	0.03	1.972	1.572	[26]
STAT2	Gastric papillary adenocarcinoma vs N	0.012	6.116	1.017	[19]
	Gastric tubular adenocarcinoma vs N	0.012	2.375	1.053	[19]
	GC vs N	8.55E-09	5.946	1.411	[22]
	Diffuse type vs N	0.005	2.81	1.389	[21]
	Mixed type vs N	0.016	2.336	1.468	[21]
	Intestinal type vs N	0.002	3.137	1.458	[21]
	GC vs N	1.42E-04	3.949	1.039	[25]
	Mixed type vs N	0.039	1.964	1.05	[25]
	Gastric adenocarcinoma vs N	2.96E-04	3.598	1.023	[25]
	Diffuse type vs N	0.001	3.223	1.013	[25]
	Intestinal type vs N	0.025	2.026	1.019	[25]
	GC vs N	0.005	2.786	1.403	[26]
	Mixed type vs N	0.017	3.09	1.642	[24]
STAT3	Mixed type vs N	6.45E-06	7.834	2.19	[24]
	Diffuse type vs N	4.08E-04	5.117	2.096	[24]
	Intestinal type vs N	2.26E-10	7.653	2.252	[24]
	GC vs N	0.002	2.909	1.203	[22]
	GC vs N	0.002	3.175	1.348	[26]
	Diffuse type vs N	0.001	3.171	1.303	[23]
	Intestinal type vs N	0.048	1.709	1.167	[23]
	Intestinal type vs N	2.20E-04	3.675	1.231	[21]
	GC vs N	0.007	2.55	1.026	[25]
	Gastric adenocarcinoma vs N	0.01	2.39	1.019	[25]
STAT4	Intestinal type vs N	0.026	2.006	1.026	[19]
	Gastric adenocarcinoma vs N	2.05E-04	3.727	1.034	[25]
	Diffuse type vs N	0.008	2.501	1.016	[25]
STAT5a	Mixed type vs N	4.53E-04	5.012	2.563	[24]
	GC vs N	0.002	3.238	1.483	[26]
	GC vs N	0.033	1.855	1.277	[22]
	Intestinal type vs N	0.047	1.727	1.247	[23]
	Diffuse type vs N	0.042	1.765	1.204	[23]
	GC vs N	0.007	2.55	1.026	[25]
	Gastric adenocarcinoma vs N	0.01	2.39	1.019	[25]
STAT5b	Mixed type vs N	5.59E-04	5.007	2.895	[24]
	Intestinal type vs N	2.63E-05	4.318	1.24	[21]
	Mixed type vs N	0.009	2.711	1.225	[21]
	Diffuse type vs N	0.017	2.262	1.166	[21]
	GC vs N	0.003	3.07	1.728	[26]
	Gastric adenocarcinoma vs N	0.043	1.723	1.017	[19]
	GC vs N	0.007	2.55	1.026	[25]
	Gastric adenocarcinoma vs N	0.014	2.239	1.019	[25]
STAT6	Gastric papillary adenocarcinoma vs N	0.012	6.122	1.017	[19]
	Gastric tubular adenocarcinoma vs N	0.012	2.376	1.053	[19]
	GC vs N	1.42E-04	3.949	1.039	[25]
	Gastric adenocarcinoma vs N	1.65E-04	3.775	1.024	[25]
	Diffuse type vs N	0.001	3.223	1.013	[25]
	Mixed type vs N	0.004	3.102	1.861	[24]
	Intestinal type vs N	0.026	1.988	1.536	[24]

### The correlation analysis between STATs and TIICs

**Figure 6:**
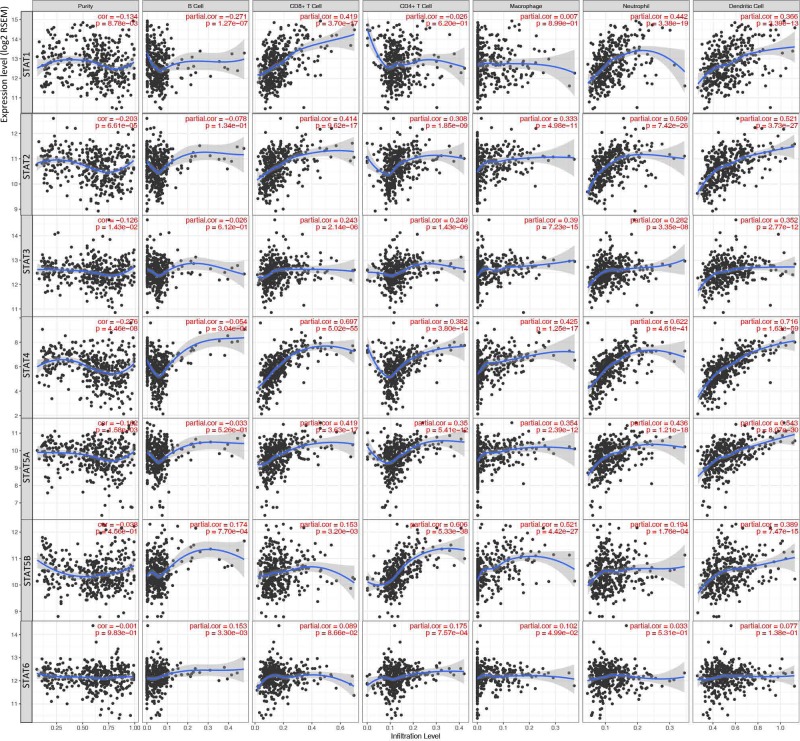
Correlation of TIICs and STATs members B cells, CD4^+^ T cells, CD8^+^ T cells, neutrophils, macrophages, and dendritic cells were compared with the STATs regarding the expression correlation.

Noteworthy, the highest correlations were found in STAT4 versus dendritic cell (correlation = 0.716, *P*=1.63e-59), and STAT4 versus CD8^+^ T cells (correlation = 0.697, *P*=5.02e-55). Meanwhile, six correlations were found above 0.5, including STAT4 versus neutrophil cell (correlation = 0.622, *P*=4.61e-41), STAT5A versus dendritic cell (correlation = 0.543, *P*=8.07e-30), STAT5B versus CD4^+^ T cell (correlation = 0.606, *P*=5.33e-38), STAT5B versus macrophage cell (correlation = 0.521, *P*=4.42e-27), STAT2 versus neutrophil cell (correlation = 0.509, *P*=7.42e-26) and STAT2 versus dendritic cell (correlation = 0.521, *P*=3.73e-27) (Figure 6).

## Discussion

STATs gene family have participated in diverse roles in carcinogenesis and tumor progression. In the present study, the prognostic roles of STATs members in GC patients were assessed through the KM plotter. High mRNA expression of STAT1, STAT2, STAT4, STAT5a, and STAT5b were significantly correlated to a favorable OS in GC patients.

Moreover, inverse prognostic values of STAT1, STAT2, and STAT6 were found between Lauren’s subtypes. Meanwhile, we also observed inverse prognostic values of STAT1 and STAT2 between the moderately and poorly differentiated subtypes. Several reasons may hold accountable. First, comparable smaller sample size in subtype analysis may be a confounding bias factor in prognosis evaluation, particularly in mixed subtype of STAT1 (*n*=25), STAT6 (*n*=25). Second, both Lauren’s classification and differentiation represent key pathological features of GC, in which molecular features are yet to be fully disclosed. Distinct prognostic values between each subtype in the present study may contribute to deeper knowledge of the pathological keys in GC. Third, the inverse prognostic values open up a new question as whether STATs may have opposite roles in each subtype.

Several studies had reported that STAT1 plays a role in gastric inflammation and tumorigenesis in mice model [27,28]. But so far, studies about STAT1 and prognosis in GC are limited. Deng *et al.* [29] indicated that STAT1 mRNA was related to favorable prognosis in GC. Our results revealed that high expression of STAT1 mRNA was associated with a better OS in all GC patients. This result is in line with previous result. Stage specifically, we observed that high mRNA expression of STAT1 indicated a better OS in stage III. Collectively, our study suggested that STAT1 may be a prognosis predictor especially for late stage and poorly differentiated GC patients.

The role of STAT2 in GC remains limited. In the current study, we found that high STAT2 mRNA level was associated with favorable OS. Similar to STAT1, high STAT2 mRNA expression was associated with better OS in poorly differentiated and stage III GC patients.

STAT3, activated by tyrosine phosphorylation in response to growth factors and cytokines, was mainly involved in the oncogenesis of several human cancers, including GC [30,31]. Previous studies showed the relationship between STAT3 mRNA expression and the prognosis in GC patients. However, controversies remained. Some studies [14,32–37] indicated that elevated STAT3 mRNA expression was associated with poor outcomes in GC patients. Nonetheless, Woo *et al.* [37] reported that the overexpression of STAT3 was correlated with favorable outcome of patients with GC. Nevertheless, Xiong *et al.* [38] and Lee *et al.* [39] showed no correlation between high STAT3 mRNA expression and OS. Interestingly, our data found there was no significant correlation between STAT3 mRNA expression and OS of GC patients. This can be attributed to different study design, race diversity, clinical stage, sample size, and cutoff definition. Moreover, Chatterjee *et al.* [34] showed that STAT3 expression was associated with poor prognosis in the intestinal subtype, consistent with our findings. Furthermore, Deng *et al.* [35] and Kim *et al.* [16] demonstrated that high STAT3 expression was significantly associated with lymph node invasion, which was consistent with our results that high STAT3 mRNA level was significant associated with unfavorable OS. The present study also indicated that STAT3 mRNA expression was correlated to poor prognosis in M0 stage GC patients. Collectively, although STAT3 showed no effect on OS, but it remained a potential prognostic predictor in subtypes of GC.

Similar to STAT2, studies about STAT4 and its prognosis in GC patients are limited. Nishi *et al.* [40] indicated that high STAT4 expression was associated with better disease free survival in GC. Our results revealed that high STAT4 mRNA expression was significantly associated with better OS for all GC patients, subtypes in HER2-positive, diffuse subtype, stage III, and lymph node-positive GC.

STAT5 is consisted of two highly homologous genes, STAT5a and STAT5b [41]. In GC, Kim *et al.* [16] reported that STAT5 had no statistical significance in the analysis of survival. However, our results suggested that high mRNA expression STAT5a and STAT5b were significantly associated with better OS.

So far, studies focusing on the prognostic value of STAT6 expression in malignancies, especially GC, remain limited. In the present study we found that high STAT6 mRNA level was not significantly associated with OS.

The present study has some limitations, including lack of experimental and clinical validations to confirm the prognostic values of STATs in GC, as well as comparably small sample size in some subtypes analysis. More studies with large sample size are warranted to validate the prognostic value of STATs family.

## Conclusion

The present study showed that high mRNA expression of STATs except STAT3 and STAT6 were significantly correlated to favorable OS in GC patients.

## Abbreviations

CI: confidence interval
GC: gastric cancer
GEO: Gene Expression Omnibus
HER2: human epidermal growth factor receptor 2
HR: hazard ratio
KM: Kaplan–Meier
OS: overall survival
PTKs: protein tyrosine kinase
STAD: stomach adenocarcinoma
STAT: signal transducers and activators of transcription genes family
TCGA: the cancer genome atlas
TIICs: tumor immune infiltrating cells
TIMER: Tumor IMmune Estimation Resource
